# A facile, versatile approach to hydroxyl-anchored metal oxides with high Cr(VI) adsorption performance in water treatment

**DOI:** 10.1098/rsos.160524

**Published:** 2016-11-23

**Authors:** Ji Ma, SiZhi Zuo-Jiang, Yunhao He, Qinglei Sun, Yunguo Wang, Wei Liu, Shuangshuang Sun, Kezheng Chen

**Affiliations:** Lab of Functional and Biomedical Nanomaterials, College of Materials Science and Engineering, Qingdao University of Science and Technology, No. 53 Zhengzhou Road, Qingdao 266042, People's Republic of China

**Keywords:** water treatment, metal oxides, adsorption, chromium

## Abstract

In this study, a facile and versatile urea-assisted approach was proposed to synthesize Chinese rose-like NiO, pinecone-like ZnO and sponge-like CoO adsorbents. The presence of urea during syntheses endowed these adsorbents with high concentration of surface hydroxyl groups, which was estimated as 1.83, 1.32 and 4.19 mmol [OH^−^] g^−1^ for NiO, ZnO and CoO adsorbents, respectively. These surface hydroxyl groups would facilitate the adsorption of Cr(vi) species (e.g. HCrO_4_^−^, Cr_2_O_7_^2−^ and CrO_4_^2−^) from wastewater by exchanging with hydroxyl protons or hydroxide ions, and hence result in extremely high maximum adsorbed amounts of Cr(vi), being 2974, 14 256 and 408 mg g^−1^ for NiO, ZnO and CoO adsorbents in the pH range of 5.02–5.66 at 298 K, respectively. More strikingly, the maximum adsorbed amounts of Cr(vi) would be greatly enhanced as the adsorbing temperature is increased, and even amount to 23 411 mg g^−1^ for ZnO adsorbents at 323 K. Based on the kinetics and equilibrium studies of adsorptive removal of Cr(vi) from wastewater, our synthetic route will greatly improve the adsorptivity of the as-synthesized metal-oxide adsorbents, and hence it will shed new light on the development of high-performance adsorbents.

## 1. Introduction

With the accelerated process of modernization, overuse of industrial chemicals, organic compounds and fossil fuels have attracted growing attention [1,2]. Abundant existence of heavy metal ions (e.g. Ba(ii), Pb(ii), Cd(iii), Cr(iii), Cr(vi), As(iii), As(v), Co(ii), Cu(ii), Ni(ii), Zn(ii) and Hg(ii)) in water has become a topic of worldwide concern as it brings many severe challenges to the public health and environmental ecosystems [3,4]. Especially, the exorbitant usage and indiscriminate disposal of these metal ions in the metallurgical industry and chemical manufacturing have resulted in serious consequences. Up to now, many methods such as coagulation and flocculation [5], membrane separation [6], chemical precipitation [7], ion exchange and adsorption [8–12] have been used for removing these heavy metal ions from water bodies. Among these methods, adsorption techniques have been proved to be very effective and attractive for the purification of wastewater [13–15].

One of the most important factors in designing an adsorbent is the understanding of its adsorption mechanism. With regard to metal-oxide adsorbents, their most accepted adsorption mechanism should be the ion-exchange process reported many times in the literature [11–13,16,17]. The surface of metal oxides in aqueous solutions is hydroxylated due to dissociative chemisorption of water molecules, and the surface hydroxyl groups adsorb heavy metal ions from solution by the exchange with hydroxyl protons or hydroxide ions [18]. In this scenario, increasing the amount of surface hydroxyl groups on metal oxides will enhance ion-exchange capacity of these oxides, and hence will improve their adsorption performance in water treatment.

Along this line, a facile and versatile solvothermal approach was proposed in this work to fabricate metal oxides (including NiO, ZnO and CoO) with a high density of surface hydroxyl groups. In water treatment experiments, Cr(vi) was chosen as the adsorbate because of its high toxicity, carcinogenicity, mutagenicity to living organisms and extreme mobility [19,20]. The effluents from certain industries were reported to contain 50–100 mg l^−1^ of Cr(vi), which is over 1000 times higher than the maximum allowed concentration of standard for wastewater discharge [21]. Based on our kinetics and equilibrium studies of adsorptive removal of Cr(vi) from wastewater, this approach greatly enhances the adsorptivity of metal-oxide adsorbents, and hence will shed new light on the development of high-performance adsorbents.

## 2. Material and methods

### 2.1. Syntheses of metal-oxide adsorbents

Analytical nickel sulfate (NiSO_4_ · 6H_2_O), zinc acetate (Zn(CH_3_COO)_2_), cobalt nitrate (Co(NO_3_)_2_ · 6H_2_O), urea (CH_4_N_2_O), 1,2-propanediol, octanol and ethylene glycol were purchased from Sinopharm Chemical Reagent Co., Ltd. (China), and used as received without further purification. Typically, 2** **mmol of NiSO_4_ · 6H_2_O, Zn(CH_3_COO)_2_, Co(NO_3_)_2_ · 6H_2_O were separately mixed with urea in a molar ratio of 1 : 1, and they were subsequently dissolved and stirred in 50 ml of 1,2-propanediol, octanol and ethylene glycol, respectively. Then these mixtures were transferred into Teflon-lined stainless-steel autoclaves with the same capacity of 100 ml for solvothermal treatment at 160°C for 24** **h. The as-obtained precipitates were repeatedly washed with deionized water and ethanol, and finally dried at 60°C for 4** **h.

### 2.2. Characterization

The XRD patterns were recorded on a powder X-ray diffractometer (Rigaku D/max-rA) equipped with a rotating anode and a Cu-K_α1_ radiation source (*λ* = 1.5406 Å) at a step width of 0.02°. Scanning electron microscope (SEM) images were collected on a field-emission scanning electron microscope (JEOL JSM-6700F). The iso-electric point (IEP) of the sample was measured by testing the zeta-potential in aqueous solution on a Zetasizer Nano ZS (Malvern Instruments). Surface charge density was determined by conductometric titration method.

### 2.3. Batch adsorption experiments

Solutions with different Cr(vi) concentrations were prepared using K_2_Cr_2_O_7_ as the source of heavy metal ions. For the adsorption kinetic study of Cr(vi), 40** **mg of adsorbent was added into 60 ml Cr(vi) solution with initial Cr(vi) concentration of 25** **mg l^−1^ for different adsorbing time. To obtain the adsorption equilibrium isotherms, 20** **mg of adsorbent was added into a set of 30 ml Cr(vi) solutions with different Cr(vi) concentrations, and vigorously stirred for 12** **h at different temperatures. No pH buffers were used during the adsorption experiments and all the equilibrium pH values were measured between 5.02 and 5.66. After adsorption, the adsorbent was immediately separated by centrifugation from Cr(vi) solutions. Subsequently, Cr(vi) concentrations in supernatant were determined by inductively coupled plasma-optical emission spectrometer (ICP-OES, Perkin-Elmer, Optima 8000). To investigate the effect of pH value on Cr(vi) adsorption, pH values of solutions [Cr(vi), 100** **mg l^−1^] before adsorbent addition were adjusted to 3, 5, 7, 9 and 11 at 298** **K by adding 1** **mol l^−1^ HCl or NaOH solutions as needed.

## 3. Results and discussion

**Figure 1.**
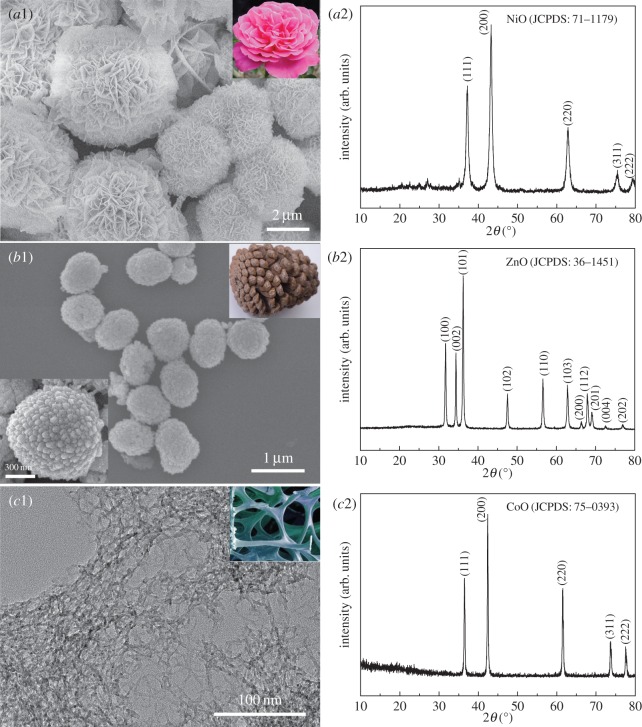
SEM images and XRD patterns of (*a*) NiO, (*b*) ZnO and (*c*) CoO adsorbents. A magnified SEM image is shown as an inset in panel *b*1, and digital photographs of Chinese rose, pinecone and sponge are shown as insets in panel *a*1, *b*1 and *c*1, respectively.

The morphology and chemical composition of the as-synthesized adsorbents are shown in figure 1, in which NiO (JCPDS No. 71–1179, figure 1*a*2), ZnO (JCPDS No. 36–1451, figure 1*b*2) and CoO (JCPDS No. 75–0393, figure 1*c*2) possess similar morphologies to commonplace Chinese rose, pinecone and sponge (insets in panel *a*1, *b*1 and *c*1), respectively. To be specific, the rose-like NiO architectures with a size of 3–6 µm are built from hundreds of self-assembled curving nanosheets with a thickness of about 20** **nm. The pinecone-like ZnO architectures are crumpled spheroidal aggregates with an average size of *ca* 900** **nm. Their particulate protuberances are not randomly assembled, as shown by a magnified SEM image in the inset of panel *b*1; instead, they are arranged along a common direction and form into the pinecone-like shape. The morphology of CoO adsorbent is akin to the sponge in our daily life, which is of open network structure assembled by interconnected nanofibres. According to the Scherrer equation, the average crystallite sizes are calculated to be *ca* 12.8, 29.5 and 32.4** **nm for rose-like NiO, pinecone-like ZnO and sponge-like CoO products, respectively, determined from (200), (111) peaks for NiO, (101), (100) peaks for ZnO, and (200), (111) peaks for CoO.

**Figure 2.**
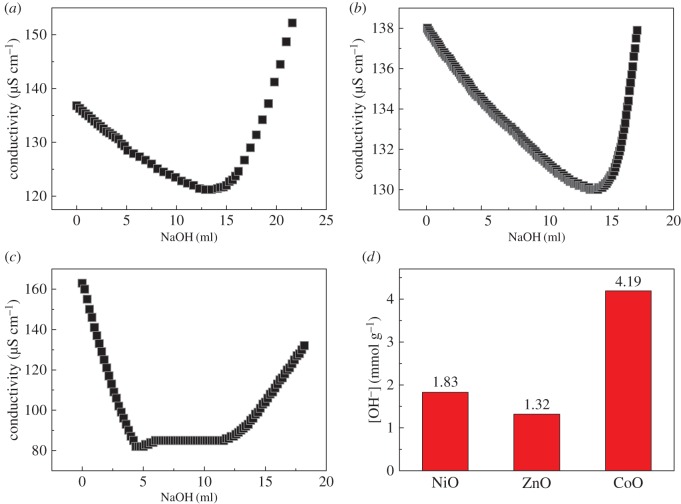
Conductometric titration of (*a*) NiO, (*b*) ZnO and (*c*) CoO adsorbents. (*d*) Surface charge density [OH^−^] of these adsorbents.

The facile syntheses of these NiO, ZnO and CoO adsorbents with well-defined morphologies share a common feature of the usage of urea during their solvothermal reactions. Generally, urea acts as a chelating agent of metal ions and in this way prevents their aggregation [22]. This peculiarity of urea can thus provide fine control of the final morphologies of these metal-oxide adsorbents. Most notably, the urea-involved hydrothermal procedure has been found to exhibit lower Lewis acidity, but higher concentration of surface hydroxyl groups [23]. To verify this point, the surface charge densities of these NiO, ZnO and CoO adsorbents were measured by conductometric titration method. In a typical measurement, 10** **mg of the adsorbent was firstly dispersed into 100 ml of de-ionized water and stirred with a magnetic bar. After adding a certain amount of hydrochloric acid, the solution was titrated with sodium hydroxide (5** **mmol l^−1^) and meanwhile the conductivity was recorded (figure 2*a*–*c*). Figure 2*d* shows that the calculated surface hydroxyl densities are 1.83, 1.32 and 4.19** **mmol [OH^−^] g^−1^ with regard to NiO, ZnO and CoO adsorbents, respectively. These values are extremely large and probably due to the usage of urea in our experiments. In this scenario, $Cr_{2}{O_{7}}^{2-}/\mathrm{HCr}{O_{4}}^{-}$ anions in wastewater will exchange with these numerous hydroxyl groups to promote the chemisorption process [11,16,17], and hence greatly improve the adsorptive capacity of these adsorbents. It should be mentioned that these surface hydroxyl densities are seemingly irrelevant to the crystallite sizes of NiO, ZnO and CoO, revealing that the assembling manner of primary particles has a critical impact on the number of their surface hydroxyl groups.

**Figure 3.**
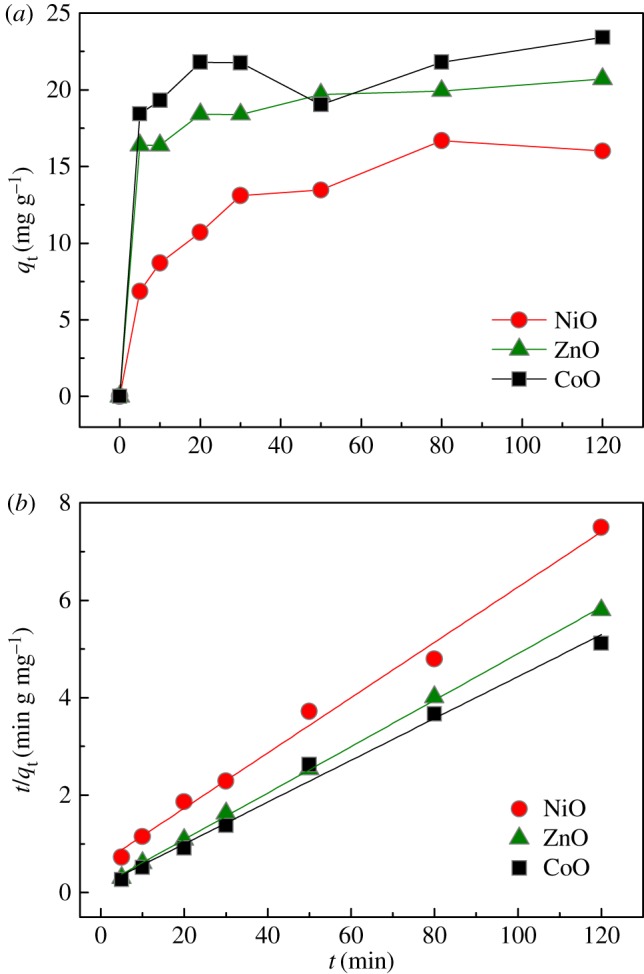
(*a*) The kinetics of Cr(VI) adsorption onto NiO, ZnO and CoO adsorbents with an initial concentration of 25 mg l^−1^ at 298 K. (*b*) Linear plots of the data in panel *a* based on the pseudo-second-order model.

Figure 3*a* shows the kinetics of Cr(vi) adsorption onto NiO, ZnO and CoO adsorbents with an initial concentration of 25** **mg l^−1^ at 298** **K. Generally, the adsorption equilibrium of Cr(vi) on these metal-oxide adsorbents was achieved within 30** **min. The pseudo-second-order kinetic model was used to fit these experimental data (figure 3*b*), and the obtained parameters are summarized and listed in table 1. The high correlation coefficients (*R*^2^) of *ca* 0.99 indicate a good description of these kinetic data by this pseudo-second-order model. More strikingly, the initial adsorption rates *h*, which can be determined by *h = kq_e_*^2^ (where *k* is the rate constant (g mg^−1^ min^−1^), *q*_e_ is the equilibrium adsorption capacity (mg g^−1^)), are above 7.0** **mg g^−1^ min^−1^ for ZnO and CoO adsorbents, which meet the requirements of ‘quick water-treatment’ in industry.

**Table 1.** Fitted parameters of Cr(VI) adsorption kinetics based on pseudo-second-order model.

adsorbents	*k* (g mg^−1^ min^−1^)	*q*_e_ (mg g^−1^)	*h* (mg g^−1^ min^−1^)	*R*^2^
NiO	0.0054	17.63	1.68	0.991
ZnO	0.0163	20.99	7.18	0.999
CoO	0.0133	23.28	7.21	0.990

**Figure 4.**
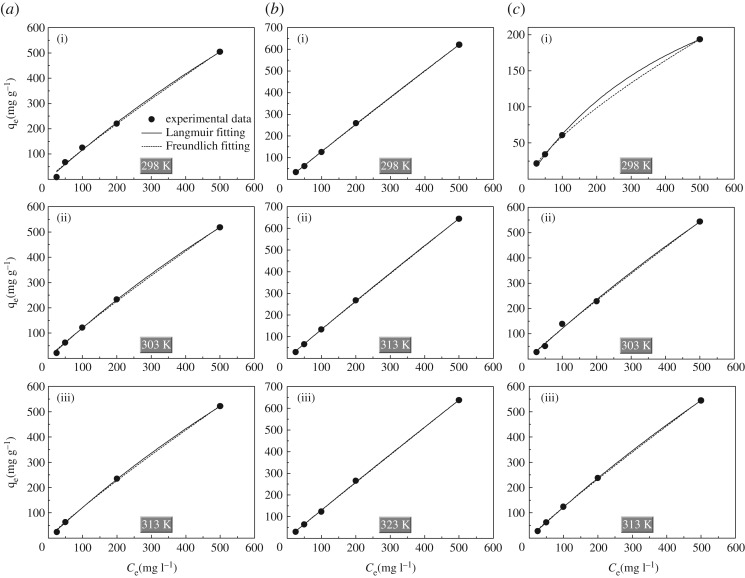
Adsorption isotherms of Cr(VI) on (*a*) NiO, (*b*) ZnO and (*c*) CoO adsorbents at different temperatures.

The adsorption isotherms of Cr(vi) on NiO, ZnO and CoO adsorbents at different temperatures are shown in figure 4. Most strikingly, the adsorbed amounts of Cr(vi) increase fast at any given Cr(vi) concentration. The Langmuir [24] and Freundlich [25] models were then used to fit these isotherm data, and the fitted parameters are summarized and listed in table 2. The high *R*^2^-values, being very close to or even equal to unity, indicate that the Langmuir and Freundlich models can both describe the adsorption isotherms in this study. The Langmuir isotherm model provides a unitless constant separation factor *R*_L_, defined by *R*_L_ = (1 + *K*_L_*C*_0_)^−1^ [26], wherein *K*_L_ is the Langmuir adsorption constant related to the affinity of binding sites (l mg^−1^) and *C*_0_ is initial concentration of Cr(vi) (mg l^−1^). The value of *R*_L_ indicates the types of Langmuir isotherm of irreversible (*R*_L_ = 0), favourable (0 <* R*_L_ < 1), linear (*R*_L_ = 1) or unfavourable (*R*_L_ > 1). By calculation, *R*_L_ is between 0 and 1 for all adsorbents at any initial Cr(vi) concentration, indicative of the favourable Langmuir isotherm in this study. According to the Langmuir fitting results, the maximum adsorbed amounts (*q_m_*) of Cr(vi) are about 2974, 14 256 and 408** **mg g^−1^ for NiO, ZnO and CoO adsorbents at 298** **K, respectively. As adsorbing temperature is elevated, the *q_m_* value progressively increases in the case of ZnO and CoO adsorbents, while it remains stable for the NiO adsorbent. The strong upward trend of *q_m_* values regarding ZnO and CoO adsorbents verifies the presence of chemisorption process (i.e. ion-exchange reaction) in water treatment. By contrast, the nearly unchanged *q_m_* values of the NiO adsorbent at any given temperature indicate the presence of other determinants, which will be discussed later. It is noteworthy that most of these *q_m_* values rival or even surpass the reported values in the literature [27–29], strongly evidencing that our versatile urea-assisted approach is remarkably advantageous in synthesizing hydroxyl-anchored metal-oxide adsorbents with high Cr(vi) adsorption performance. On the other hand, the Freundlich model describes the adsorption on an energetically heterogeneous surface on which the adsorbed molecules are interactive. The Freundlich constants *K*_F_ ((mg g^−1^)(l mg^−1^)^1/*n*^) and *n* (unitless) are related to the adsorbed amount and adsorption affinity, respectively. The values of 1/*n* are less than 1 for all Cr(vi) adsorbents, indicating the degree of nonlinearity between the solution concentration and amount of Cr(vi) ions adsorbed [30].

**Table 2.** Fitted parameters of Cr(VI) adsorption isotherms for NiO, ZnO and CoO adsorbents at different temperatures.

		Langmuir model			Freundlich model		
adsorbents	*T* (K)	*K*_L_ (l mg^−1^)	*q_m_* (mg g^−1^)	*R^2^*	*K*_F_ ((mg g^−1^) (l mg^−1^)^1/*n*^)	*n*	*R^2^*
NiO	298	0.000408	2973.70	0.995	1.752	1.097	0.994
	303	0.000424	2961.71	0.999	1.827	1.100	0.998
	313	0.000426	2968.88	0.999	1.865	1.103	0.998
ZnO	298	0.0000911	14 255.88	1.000	1.404	1.020	1.000
	313	0.0000910	14 807.61	1.000	1.455	1.020	1.000
	323	0.0000560	23 411.79	0.999	1.353	1.009	0.999
CoO	298	0.0018	408.37	0.998	2.006	1.360	0.999
	303	0.000296	4208.80	0.996	1.676	1.075	0.996
	313	0.000321	3936.82	1.000	1.719	1.079	0.999

**Figure 5.**
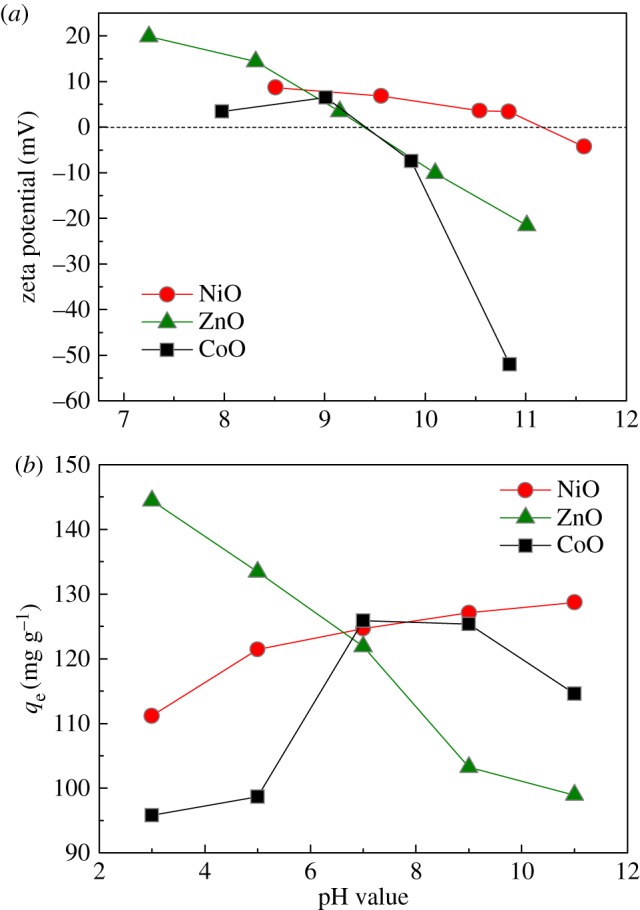
(*a*) pH-dependent zeta potential values of NiO, ZnO and CoO adsorbents in aqueous solution at 298 K. (*b*) Effect of pH values on the equilibrium adsorption capacity for these adsorbents with initial Cr(VI) concentration of 100 mg l^−1^ at 298 K.

To seek deep insight into the extremely high adsorptive capacity of these hydroxyl-anchored metal-oxide adsorbents, IEP values were determined by measuring pH-dependent zeta-potential values in aqueous solution. It is clearly observed from figure 5*a* that the IEP values can be estimated as 11.2, 9.4, 9.4 for NiO, ZnO, CoO adsorbents, respectively. The pH dependence of their equilibrium adsorption capacity is shown in figure 5*b*. As is known, Cr(vi) exists mainly in the soluble forms of both $\mathrm{HCr}{O_{4}}^{-}$ and $Cr_{2}{O_{7}}^{2-}$ at pH from 2.0 to 6.5 [20,26], and $\mathrm{HCr}{O_{4}}^{-}$ and $Cr_{2}{O_{7}}^{2-}$ anions account for about 80% and 20%, respectively, in the pH range of 2–5 [31]. As the pH value increases above 6.5, Cr(vi) exists mainly in soluble form of $\mathrm{Cr}{O_{4}}^{2-}$ [26]. Based on the above discussion, when the pH value is below the IEP values of these three adsorbents, the positive surface charge would electrostatically attract the Cr(vi) species ($\mathrm{HCr}{O_{4}}^{-}$, $Cr_{2}{O_{7}}^{2-}$ and $\mathrm{Cr}{O_{4}}^{2-}$) to enhance the adsorptive capacity of the adsorbent (e.g. ZnO case in figure 5*b*); alternatively, the excess H^+^ cations in solution may also lower the surface hydroxyl site densities on the adsorbent, and hence reduce the ion-exchange ability and adsorptive capacity of the adsorbent (e.g. NiO and CoO cases in figure 5*b*). At high pH values above the IEP values, the deprotonation of adsorbent's surface can facilitate the electrostatic repulsion between the adsorbent and Cr(vi) species (mainly $\mathrm{Cr}{O_{4}}^{2-}$) to reduce its adsorptive capacity (e.g. ZnO and CoO case in figure 5*b*).

**Figure 6.**
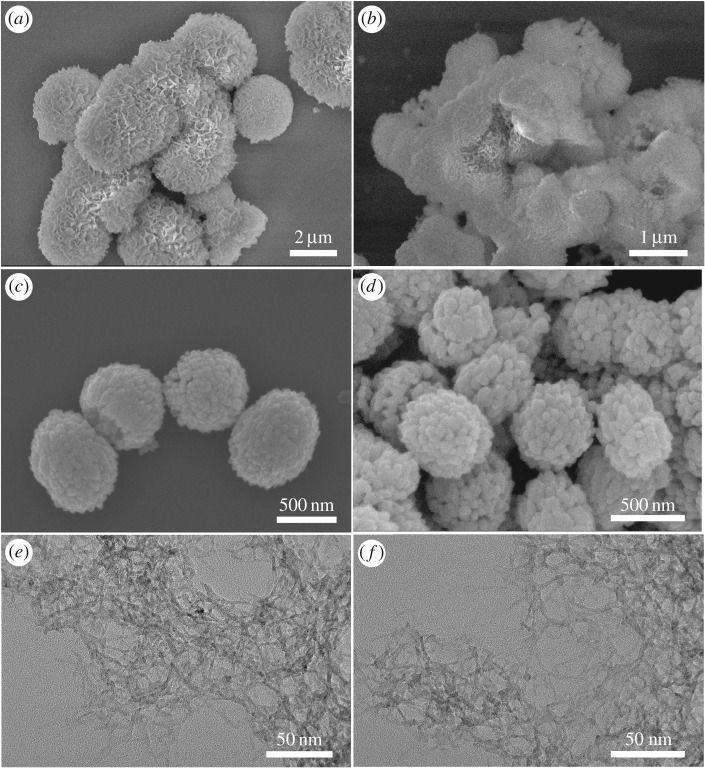
SEM images of (*a*,*b*) NiO, (*c*,*d*) ZnO, and TEM images of (*e*,*f*) CoO adsorbents after Cr(VI) adsorption (panels *a*,*c*,*e*) and desorption (panels *b*,*d*,*f*).

**Figure 7.**
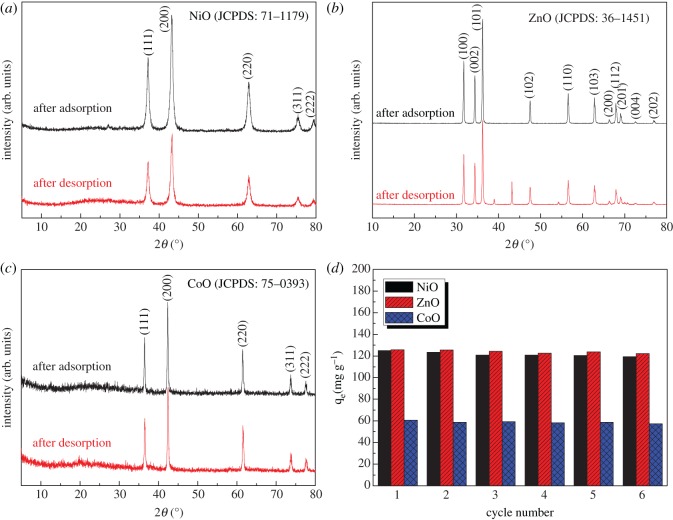
XRD patterns of (*a*) NiO, (*b*) ZnO and (*c*) CoO adsorbents after Cr(VI) adsorption and desorption. (*d*) Cr(VI) equilibrium adsorption on these adsorbents with initial Cr(VI) concentration of 100 mg l^−1^ in six successive adsorption–desorption cycles at 298 K.

Although the NiO, ZnO and CoO adsorbents exhibit different pH-dependent adsorption behaviours, their facile and versatile syntheses and high adsorptive capacity for Cr(vi) ions are most attractive in practical water treatment. Before that, these metal-oxide adsorbents have to be evaluated in at least two aspects. In the first place, desorption and reusability of these adsorbents should be investigated. In our desorption procedure, the NiO, ZnO and CoO adsorbents after Cr(vi) adsorption were firstly centrifuged from solution. After that, 1** **mol l^−1^ NaOH aqueous solution was used to convert the adsorbed Cr(vi) species to soluble Na_2_CrO_4_. Finally, these adsorbents were separated by centrifugation, washed successively with distilled water and absolute ethanol, and dried in the air at 60°C for 4** **h. Figure 6 shows the morphologies of these adsorbents after Cr(vi) adsorption and desorption. Obviously, the particle size and morphology of these adsorbents are virtually unchanged, and their chemical compositions (figure 7*a*–*c*) are also quite similar to their counterparts before Cr(vi) adsorption. This excellent retention of morphology, size and composition is of great benefit to their reusability. Figure 7*d* shows the equilibrium adsorptive experiments at 298** **K for six cycles of desorption–adsorption process. It is obvious that the equilibrium adsorption capacity renders virtually the same values, manifesting the good reusability of our adsorbents in water treatment.

**Figure 8.**
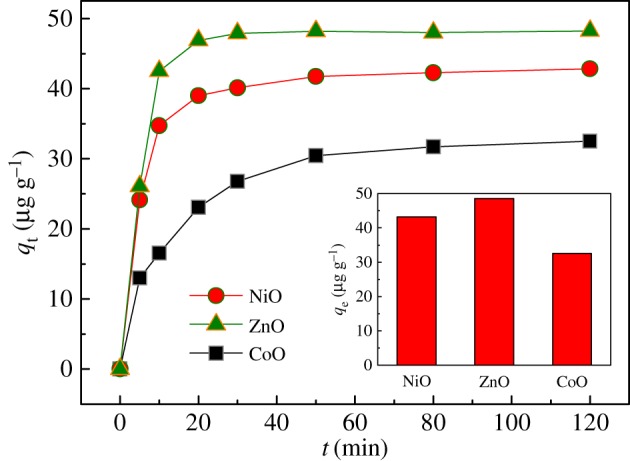
The kinetics and equilibrium (inset) of Cr(VI) adsorption on NiO, ZnO and CoO adsorbents with an initial Cr(VI) concentration of 50 µg l^−1^ at 298 K.

Additionally, the detection limit (or sensitivity) of these adsorbents is of fundamental importance in their applications. Given that the maximum contaminant level of chromium in domestic water supplies has been set by WHO as 50** **μg l^−1^ [12], thus a set of solutions with initial Cr(vi) concentration of 50** **μg l^−1^ were used to perform the kinetics and equilibrium adsorption study once again. It is clear from figure 8 that all of these adsorbents can still exhibit obvious adsorptive performance in 50** **μg l^−1^ of Cr(vi) solution, revealing that their detection limit is much lower than 50** **μg l^−1^. This is conducive to their practical water purification. Notably, the inset of figure 8 shows CoO adsorbent, being of the highest surface hydroxyl densities (figure 2*d*), exhibits the least adsorption capacity for low concentration Cr(vi) solutions. This adsorption behaviour differs significantly from the case of 100** **mg l^−1^, and can be explained by considering the adsorption mechanism. At first, the negatively charged $Cr_{2}{O_{7}}^{2-}$ or $\mathrm{HCr}{O_{4}}^{-}$ ions transfer to the periphery of adsorbents by electronic attraction, and then the enriched $Cr_{2}{O_{7}}^{2-}/\mathrm{HCr}{O_{4}}^{-}$ ions will exchange with the hydroxyl groups [12] on the adsorbent surfaces to promote the adsorption process. However, if the surface hydroxyl density is extremely high in the CoO case, the initial electrostatic adsorption process is greatly inhibited, and therefore the subsequent ion exchange process is restrained. This means the proper amount of surface hydroxyl groups will be of great benefit to high adsorption capacity.

## 4. Conclusion

In summary, Chinese rose-like NiO, pinecone-like ZnO and sponge-like CoO adsorbents were synthesized by a facile and versatile urea-assisted solvothermal procedure. Their surface hydroxyl densities were determined to be 1.83, 1.32 and 4.19** **mmol [OH^−^] g^−1^ for NiO, ZnO and CoO adsorbents, respectively. The presence of urea endowed the hydrothermal procedure with lower Lewis acidity, but higher concentration of surface hydroxyl groups, which would be conducive to the ion-exchange process between the adsorbents and Cr(vi) species in wastewater. The maximum adsorbed amounts of Cr(vi) were as high as 2974, 14 256 and 408** **mg g^−1^ for NiO, ZnO and CoO adsorbents at 298** **K, respectively. Most notably, these adsorbed amounts would be greatly enhanced as increasing the adsorbing temperature. It is anticipated that this work will shed new light on the development of high-performance adsorbents.

## Data accessibility

All data are provided in the main text.
